# Antennal transcriptome and expression analyses of olfactory genes in the sweetpotato weevil *Cylas formicarius*

**DOI:** 10.1038/s41598-017-11456-x

**Published:** 2017-09-11

**Authors:** Shu-Ying Bin, Meng-Qiu Qu, Xin-Hua Pu, Zhong-Zhen Wu, Jin-Tian Lin

**Affiliations:** Institute for Management of Invasive Alien Species, 314 Yingdong teaching building, Zhongkai University of Agriculture and Engineering, Guangzhou, 510225 P.R. China

## Abstract

The sweetpotato weevil, *Cylas formicarius* (Fabricius), is a serious pest of sweetpotato. Olfaction-based approaches, such as use of synthetic sex pheromones to monitor populations and the bait-and-kill method to eliminate males, have been applied successfully for population management of *C. formicarius*. However, the molecular basis of olfaction in *C. formicarius* remains unknown. In this study, we produced antennal transcriptomes from males and females of *C. formicarius* using high-throughput sequencing to identify gene families associated with odorant detection. A total of 54 odorant receptors (ORs), 11 gustatory receptors (GRs), 15 ionotropic receptors (IRs), 3 sensory neuron membrane proteins (SNMPs), 33 odorant binding proteins (OBPs), and 12 chemosensory proteins (CSPs) were identified. Tissue-specific expression patterns revealed that all 54 ORs and 11 antennal IRs, one SNMP, and three OBPs were primarily expressed in antennae, suggesting their putative roles in olfaction. Sex-specific expression patterns of these antenna-predominant genes suggest that they have potential functions in sexual behaviors. This study provides a framework for understanding olfaction in coleopterans as well as future strategies for controlling the sweetpotato weevil pest.

## Introduction

Olfaction plays an essential role in the life cycle of insects that use a wide range of environmental chemical cues to locate and evaluate food, mates, and egg-laying sites as well as to avoid predators and other dangers. Olfaction is therefore an important research field in insect biology^1^. Insect odor reception and signal transduction occurs in the dendritic membrane of olfactory sensory neurons (OSNs) in the antennae^2, 3^. The key molecular components are a diverse array of odorant receptors (ORs), ionotropic receptors (IRs), and odorant-binding proteins (OBPs)^1^. After entering the sensillum lymph through cuticular pores, odorant molecules are recognized, bound, and transported by OBPs or potential chemosensory proteins (CSPs) across the lymph to ORs in the dendritic membrane of OSNs. The OSNs are then activated and generate an electrical signal that is processed and transmitted to higher-order neural centers^1, 4, 5^. Sensory neuron membrane proteins (SNMPs)^6–8^ and ionotropic receptors (IRs)^9–14^ have also been reported in OSNs with acquired olfactory functions, as well as particular gustatory receptors (GRs) responding to carbon dioxide^15, 16^.

Coleoptera (beetles and weevils) is the largest and most diverse order of insects on earth, representing almost 40 percent of all described insect species (https://www.britannica.com/animal/beetle). The order contains a large number of important pests of agriculture, forestry and stored products worldwide. Sex and aggregation pheromones are critical in communication among individuals within a coleopteran species for mating and locating host plants^17, 18^.

Olfactory-related genes have been identified from several Coleopteran species, including *Tribolium castaneum* ^19, 20^, *Megacyllene caryae* ^21^, *Ips typographus* ^22^, *Dendroctonus ponderosae* ^22^, *Agrilus planipennis* ^23^, *Batocera horsfieldi* ^24^, *Anomala corpulenta* ^25, 26^, *Dendroctonus valens* ^27^, *Tenebrio molitor* ^28^, *Colaphellus bowringi* ^29^, *Ambrostoma quadriimpressum* ^30^, *Rhynchophorus ferrugineus* ^31^, *Phyllotreta striolata* ^32^, *Anoplophora glabripennis* ^33^, *Brontispa longissima* ^34^. To date, identifying functional olfactory molecules in Coleoptera is limited in a single species. In *M. caryae*, three ORs (McarOR3, McarOR20, and McarOR5) were functionally characterized that they were tuned respectively to three aggregation pheromone components ((S)-2-methyl-1-butanol, (2S, 3R)-2, 3-hexanediol and 2-phenylethanol)^21^.

The sweetpotato weevil, *Cylas formicarius* (Fabricius) (Coleoptera: Brentidae), is a serious pest of sweetpotato (*Ipomea batatans*)^35^. *C. formicarius* damages sweet potatoes both in the field and in storage^36^ and it is a quarantine pest. Owing to the cryptic feeding habits of the larvae and the nocturnal activities of the adults, *C. formicarius* is difficult to control using conventional chemical insecticides. Due to the life-history characteristics of *C. formicarius*, chemical control has typically been achieved with residual insecticides such as spinosad and azadirachtin. However, this management approach has led to control failures due to development of insecticide resistance^37^. Olfaction-based approaches, using synthetic sex pheromones and host volatiles to interfere with the pests’ ability to find suitable mates and hosts, have been used successfully in “push-pull” control strategies^38^. The male *C. formicarius* is very sensitive to the pheromone active component (Z)-3-dodecen-1-ol (E)-2-butenoate, released by virgin females^39, 40^. Pheromone-baited traps used for population monitoring and mass trapping can provide effective control of this pest^41, 42^. However, the molecular mechanisms involved with *C. formicarius* finding mates or host plants are still unknown. Hence, a detailed knowledge of olfaction in insects is imperative.

The goal of the present study was to identify the genes involved in olfaction from the male and female antennae of *C. formicarius* using high-throughput sequencing. The expression profiles in different tissues were studied using semi-quantitative RT-PCR and real-time quantitative-PCR and their putative olfactory functions are proposed. Evolutionary relationships with other Coleoptera olfaction genes are discussed.

## Results

### Illumina sequencing

A total of 51,183,400 and 48,433,914 raw reads were obtained from male and female antennae cDNA libraries of *C. formicarius*, respectively. Trimming adaptor sequences, eliminating low quality reads and contaminating sequences produced 51,025,928 and 48,238,192 clean reads from male and female antennae respectively. After a combined assembly, the two datasets resulted in 66,531 unigenes with a mean length of 1,384 bp, an N50 of 2,924 bp, and an N90 of 506 bp (see Supplementary Table S1).

### Functional annotation of assembled unigenes

**Figure 1:**
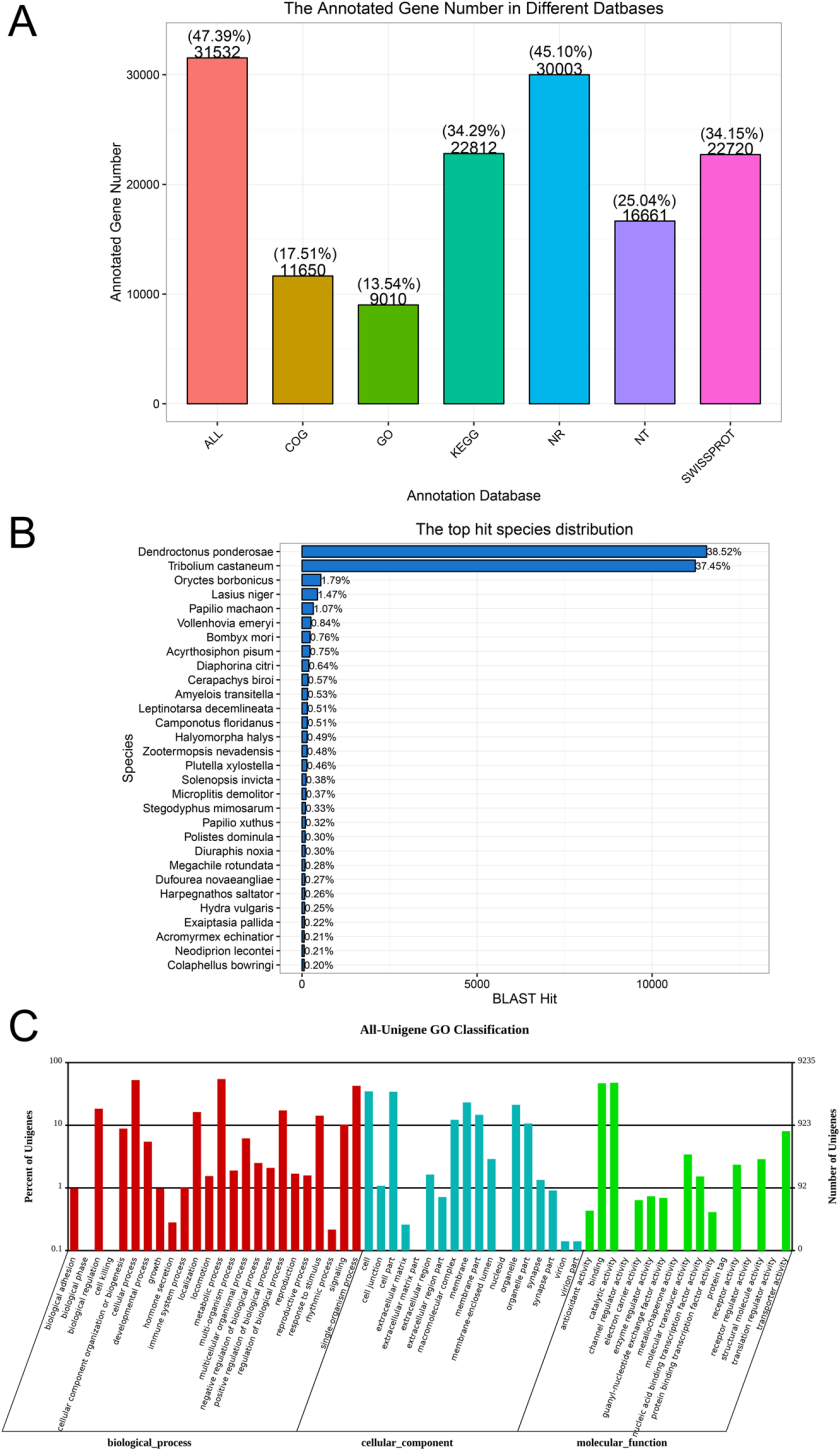
(**A**) Unigenes annotated through the different databases. (**B**) Percentage of homologous hits of the *C. formicarius* transcripts to other insect species. The *C. formicarius* transcripts were searched by BLASTx against the non-redundant protein database with a cutoff E-value of 10^−5^. (**C**) Gene ontology (GO) classification of the *C. formicarius* transcripts with Blast2GO program. One unigene was annotated to more than one GO term.

Annotation was conducted by BLASTx and BLASTn program with the E-value cut-off of 10^−5^, 31,532 (47.39%) unigenes were annotated by at least one of the databases: 30,003 (45.10%) unigenes were annotated by the NCBI-Nr database, 16,661 (25.04%) unigenes by the NCBI-Nt database, 22,720 (34.15%) by SwissPort, 9,010 (13.54%) by GO, 11,650 (17.51%) by the COG database, 22,812 (34.29%) by KEGG (Fig. 1A). BLASTx homology searches in the NCBI-Nr database showed that *C. formicarius* antennal transcriptomes had a best blast match to coleopteran sequences, primarily the mountain pine beetle *D. ponderosae*, (38.52%) and the red flour beetle *T. castaneum* (37.45%) (Fig. 1B). With the GO classification, all unigenes in *C. formicarius* antennal transcriptomes were classified into 3 functional categories: molecular function, biological process, and cellular component (Fig. 1C). In molecular function, the most abundant transcripts in the antennae were linked to binding and catalytic activity. In biological process, the most represented biological processes were cellular, metabolic, and single-organism processes. In the cellular component terms, cell, cell part, and membrane constituted the most abundant categories. These GO assignments are in accordance with those reported previously for dipteran^43–45^, lepidopteran^46–48^, and other coleopteran antennal transcriptomes^22, 27, 33^.

### Gene families associated with odorant detection

Gene families involved in odorant detection, including ORs (54 transcripts), GRs (11 transcripts), IRs (15 transcripts), SNMPs (3 transcripts), OBPs (26 transcripts) and CSPs (12 transcripts) were identified in male and female *C. formicarius* antennal transcriptomes. Information of candidate ORs, GRs, IRs, SNMPs, OBPs, CSPs and housekeeping genes including the gene name, unigene sequences, lengths, predicted protein sequences and the annotation in NCBI-Nr database, predicted protein domains and expression abundance are listed in the Supplementary Dataset.

### Identification of odorant receptors

**Figure 2:**
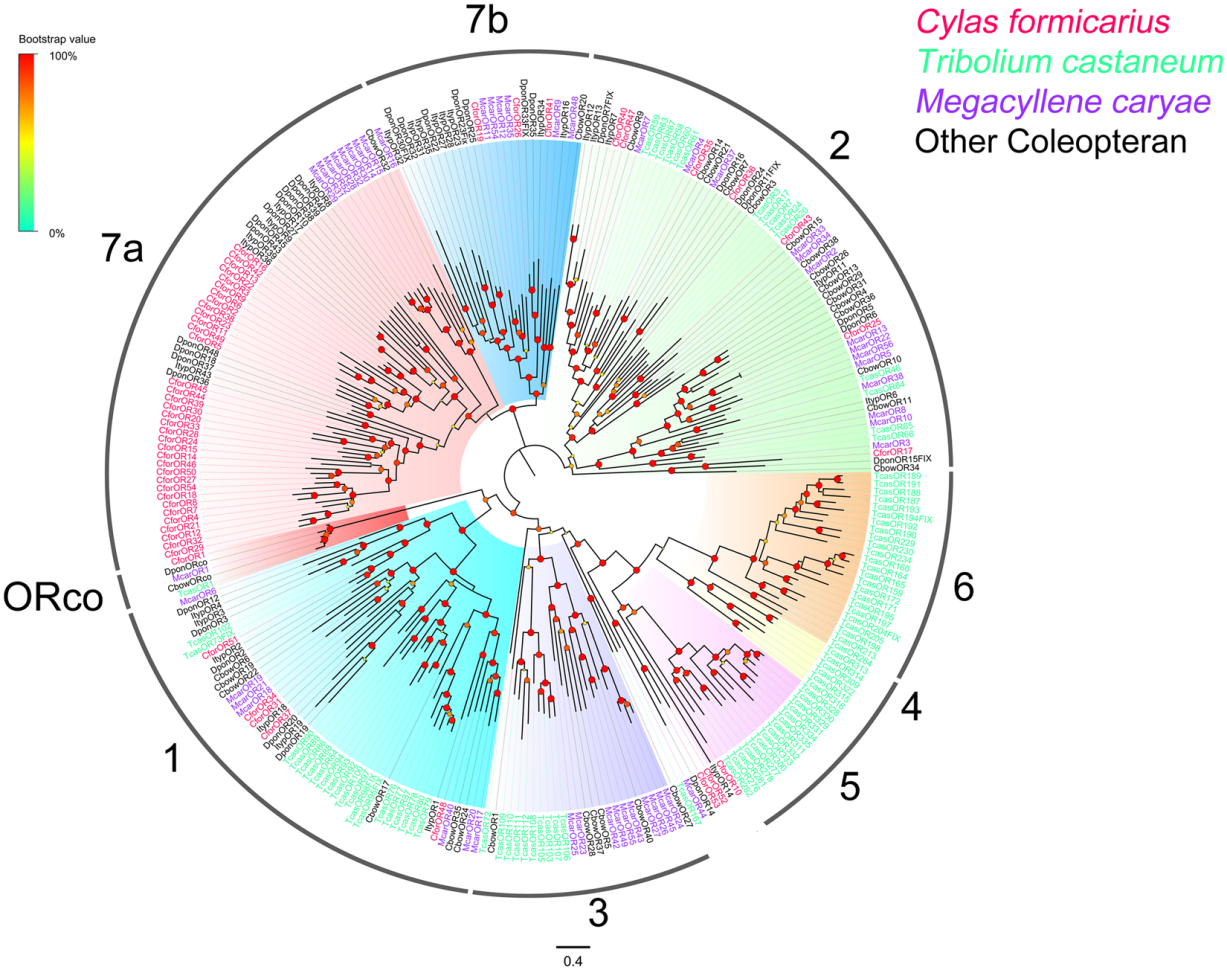
Phylogenetic analysis of ORs. Species abbreviations: Cfor, *Cylas formicarius*; Tcas, *Tribolium castaneum*; Mcar, *Megacyllene caryae*; Ityp, *Ips typographus*; Dpon, *Dendroctonus ponderosae*; Cbow, *Colaphellus bowringi*. Branch support (circles at the branch nodes) was estimated using an approximate likelihood ratio test based on the scale indicated at the top left. Bars indicate branch lengths in proportion to amino acid substitutions per site.

Bioinformatic analysis of the *C. formicarius* male and female antennal transcriptomes identified 54 candidate OR transcripts, which were classified as belonging to the 7-transmembrane receptors superfamily. Of these, 40 represented full-length open reading frames (ORF), based upon the presence of predicted start and stop codons and 5′ and 3′ untranslated regions (UTR). The highly conserved co-receptor (Orco) was identified in the *C. formicarius* transcriptomes, sharing 84.41% amino acid sequence identity with *C. bowringi* Orco. The specific ORs (53 ORs) in *C. formicarius* antennae shared low identity (19.26% to 59.52% identity) with known coleopteran ORs in the NCBI (see Supplementary Dataset). A phylogenetic analysis was conducted using a data set containing the sequences of all 54 ORs in *C. formicarius* and other coleopteran ORs from antennal transcriptomes or expressed in antenna (Fig. 2). Phylogenetic analysis demonstrated the clustering of CforORs with the previously defined coleopteran OR subgroups 1, 2, 7a and 7b, as well as the Orco subgroup^19^, and the vast majority of CforORs were assigned to OR subgroup 7a (65%, 35 ORs).

**Figure 3:**
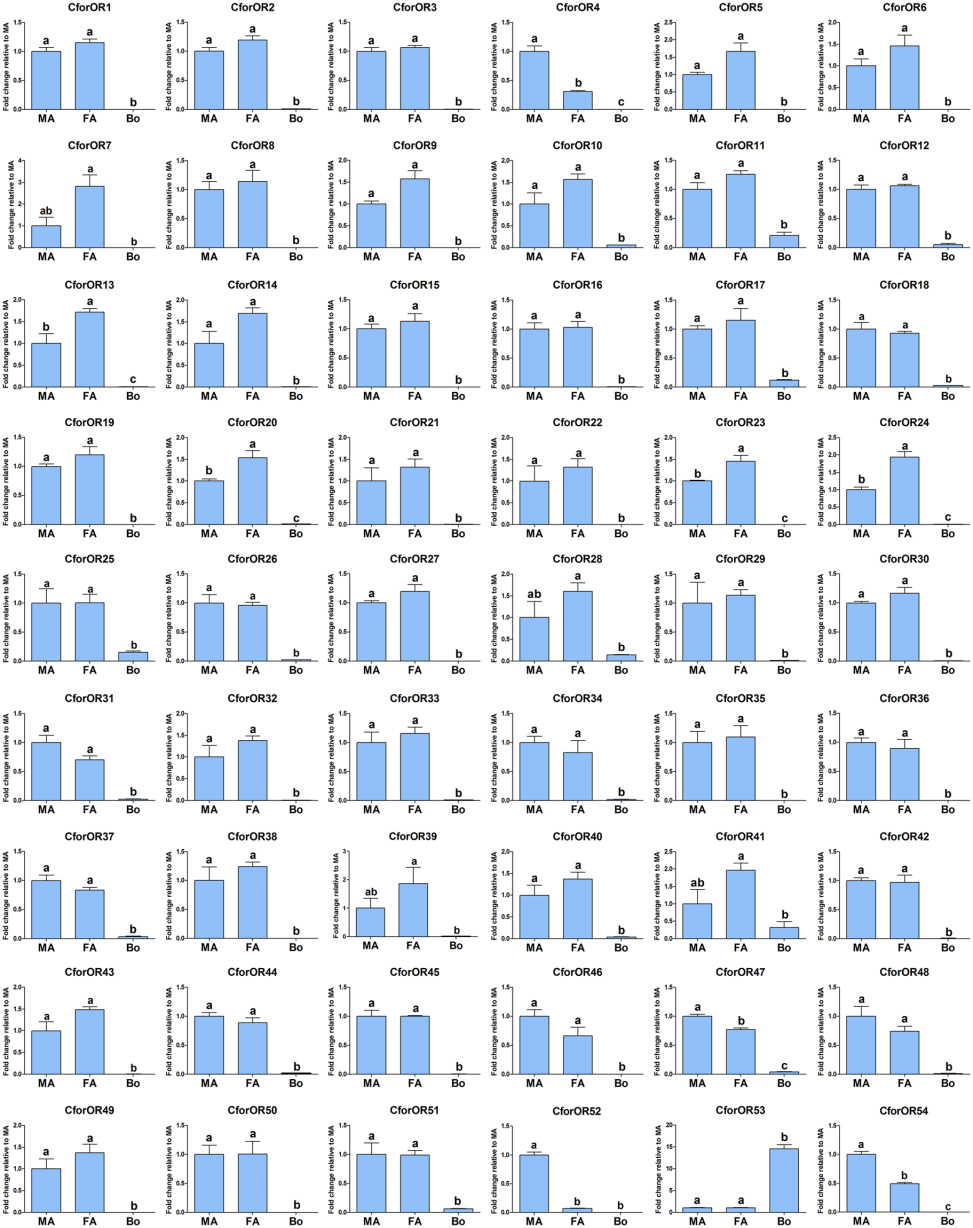
Relative expression levels of putative *C. formicarius* ORs in the male and female antennae, and whole insect body, using qPCR. Abbreviations: MA, female antennae; FA, female antennae; Bo, whole insect body without antennae. The relative expression level is indicated as mean ± SE (n = 3). Standard error is represented by the error bar, and different letters indicate significant differences between tissues (p < 0.05, ANOVA, HSD).

As expected, the CforOrco gene exhibited the highest abundance both in male and female antennae (male RPKM: 645.76; female RPKM: 703.3). The transcriptional profiles of CforOR genes were characterized using qPCR, and the results revealed that all of the 53 CforORs except CforOR53 displayed predominately antenna linked or otherwise biased expression levels. Although we did not identify sex-specific genes in these *C. formicarius* olfactory receptors, there were four (CforOR4, 47, 52, and 54) with significantly higher expression in the male antennae and four (CforOR13, 20, 23 and 24) with significantly higher expression in the female antennae, respectively (Fig. 3). Additionally, qPCR data for five CforORs (4, 13, 23, 52 and 54) mirrored the RNA-seq data.

### Gustatory receptors

**Figure 4:**
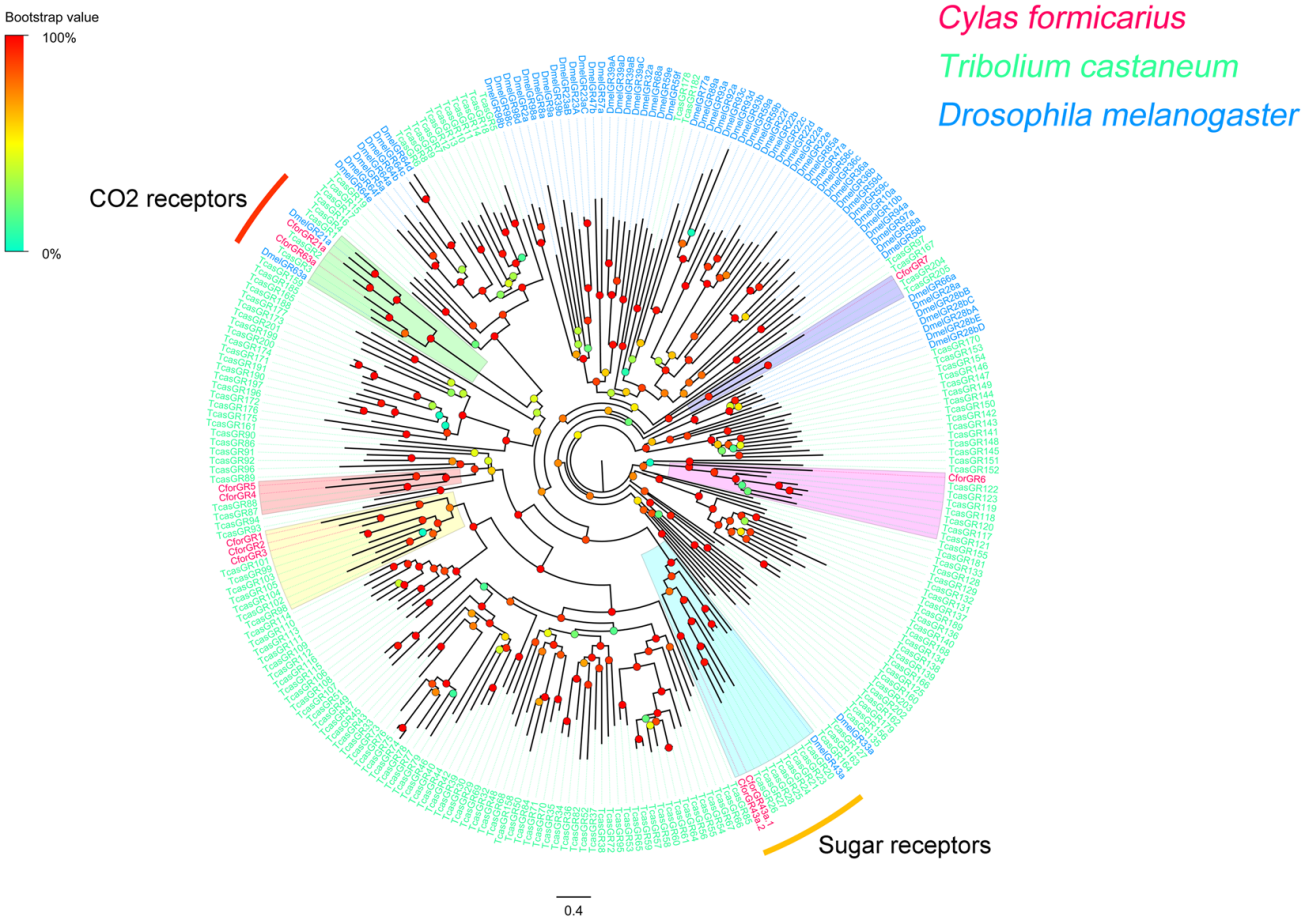
Phylogenetic analysis of GRs. Species abbreviations: Cfor, *Cylas formicarius*; Tcas, *Tribolium castaneum*; Dmel, *Drosophila melanogaster*. Branch support (circles at the branch nodes) was estimated using an approximate likelihood ratio test based on the scale indicated at the top left. Bars indicate branch lengths in proportion to amino acid substitutions per site.

In the antennal transcriptomes, 11 candidate GR transcripts were identified. Most candidate CforGRs were partial fragments (only CforGR63a represent full-length proteins), encoding overlapping but distinct sequences. This establishes the proteins as being fragments of independent genes. A phylogeny was built with 11 CforGRs, and GRs from *T. castaneum*, and *D. melanogaster* (Fig. 4). The CforGRs grouped with their presumed Drosophila orthologues, which have been shown to have roles in carbon dioxide detection (GR21a and GR63a)^16, 49^. Two CforGRs clustered within known Drosophila sugar (DmelGR43a) receptors^50, 51^, and the remaining CforGRs were assigned to different phylogenetic group with *T. castaneum* GRs. In addition, RPKM results showed low-level expression of all CforGRs in both male and female antennae (RPKM: 0~5.26).

### Ionotropic receptors

**Figure 5:**
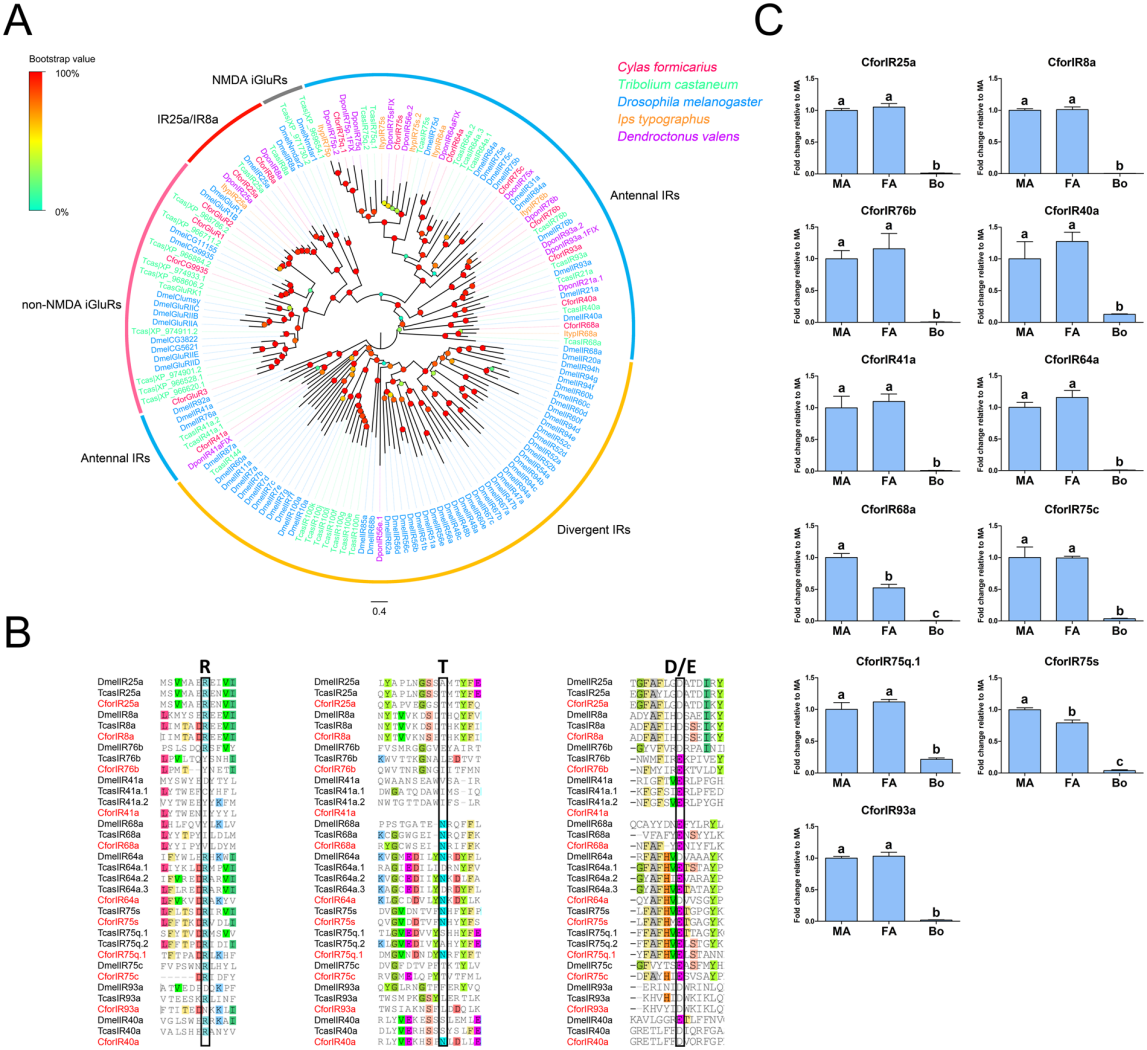
(**A**) Phylogenetic analysis of iGluRs/IRs. Species abbreviations: Cfor, *Cylas formicarius*; Tcas, *Tribolium castaneum*; Dmel, *Drosophila melanogaster*; Ityp, *Ips typographus*; Dpon, *Dendroctonus ponderosae*. Branch support (circles at the branch nodes) was estimated using an approximate likelihood ratio test based on the scale indicated at the top left. Bars indicate branch lengths in proportion to amino acid substitutions per site. (**B**) Excerpts from the amino acids alignment showing the predicted iGluRs/IRs binding domains. (**C**) Relative expression levels of putative *C. formicarius* antennal IRs in the male and female antennae, and whole insect body. Abbreviations: MA, female antennae; FA, female antennae; Bo, whole insect body without antennae. The relative expression level is indicated as mean ± SE (n = 3). Standard error is represented by the error bar, and different letters indicate a significant difference between tissues (p < 0.05, ANOVA, HSD).

Fifteen candidate iGluRs/IRs transcripts were identified from the antennal transcriptomes, which were predicted to encode ligand-gated cation channels (S1 and S2) with three transmembrane domains (M1, M2, and M3) or portions of domains. Among these, nine iGluRs/IRs represented full-length ORFs encoding more than 540 amino acids. All identified iGluRs/IRs in *C. formicarius* clustered with their orthologs from *T. castaneum*, *I. typographus*, *D. ponderosae* and *D. melanogaster* and were assigned to three phylogenetic groups including non-N-Methyl-D-aspartic acid (NMDA) iGluRs, IR co-receptors (IR25a/IR8a), and antennal IRs (Fig. 5A). With the antennal IRs, two IR co-receptors (CforIR25a and CforIR8a), and CforIR75c retained all key amino acids of predicted glutamate binding domains (R, T and D/E)^52^, and one or more of these positions were absent in other antennal IR candidates indicating variable ligand binding properties (Fig. 5B).

The RPKM results showed that CforIR93a (male RPKM: 57.49; female RPKM: 78.79) displayed the highest expression levels, followed by CforIR76b. The qPCR results for *C. formicarius* antennal IRs revealed that all of the 11 candidates displayed predominately antenna linked expression levels. There were two (CforIR68 and 75 s) and two (CforIR64 and 93a) with significantly higher expression in the male and female antennae, respectively (Fig. 5C). These qPCR results matched the FPKM data for sex differences.

### Sensory neuron membrane proteins

**Figure 6:**
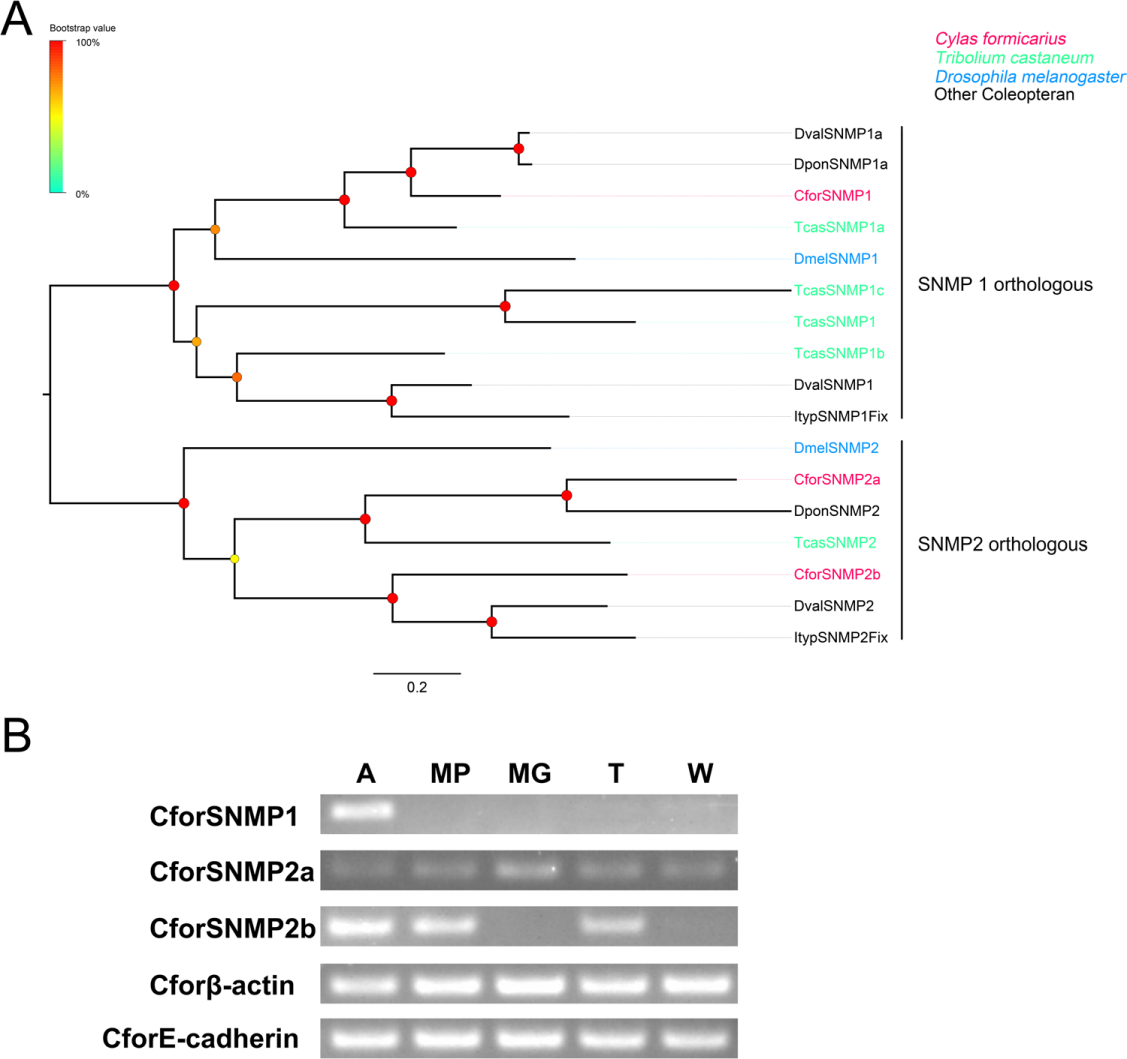
(**A**) Phylogenetic analysis of SNMPs. Species abbreviations: Cfor, *Cylas formicarius*; Tcas, *Tribolium castaneum*; Dmel, *Drosophila melanogaster*. Ityp, *Ips typographus*; Dpon, *Dendroctonus ponderosae*; Dval, *Dendroctonus valens*. Branch support (circles at the branch nodes) was estimated using an approximate likelihood ratio test based on the scale indicated at the top left. Bars indicate branch lengths in proportion to amino acid substitutions per site. (**B**) Transcriptional profiles of putative *C. formicarius* SNMPs in different body parts as determined using semi-quantitative RT-PCR. Two housekeeping genes, β-actin (Cforβ-actin) and E-cadherin (CforE-cadherin), were used as internal references to test the integrity of each cDNA template. Abbreviations: A: antenna; MP, mouthparts; MG, midgut; T, tarsus; W, wing.

Three candidate SNMP transcripts were identified that matched the CD36 family, with a full ORF and two transmembrane domains. Phylogenetic analysis showed that CforSNMP1 was clustered with the homologous SNMP1 group from other insect species, while CforSNMP2a and CforSNMP2b clustered with SNMP2 group homologs from other insects (Fig. 6A).

The SNMP1 subfamilies, CforSNMP1 with a relatively high abundance of transcripts in the antennal transcriptome of both sexes, appears to be expressed at high levels (male RPKM: 640.23; female RPKM: 457.03) in *C. formicarius* antennae. Semi-quantitative RT-PCR analysis revealed that CforSNMP1 was exclusively expressed in antennae (Fig. 6B).

### Odorant binding proteins

**Figure 7:**
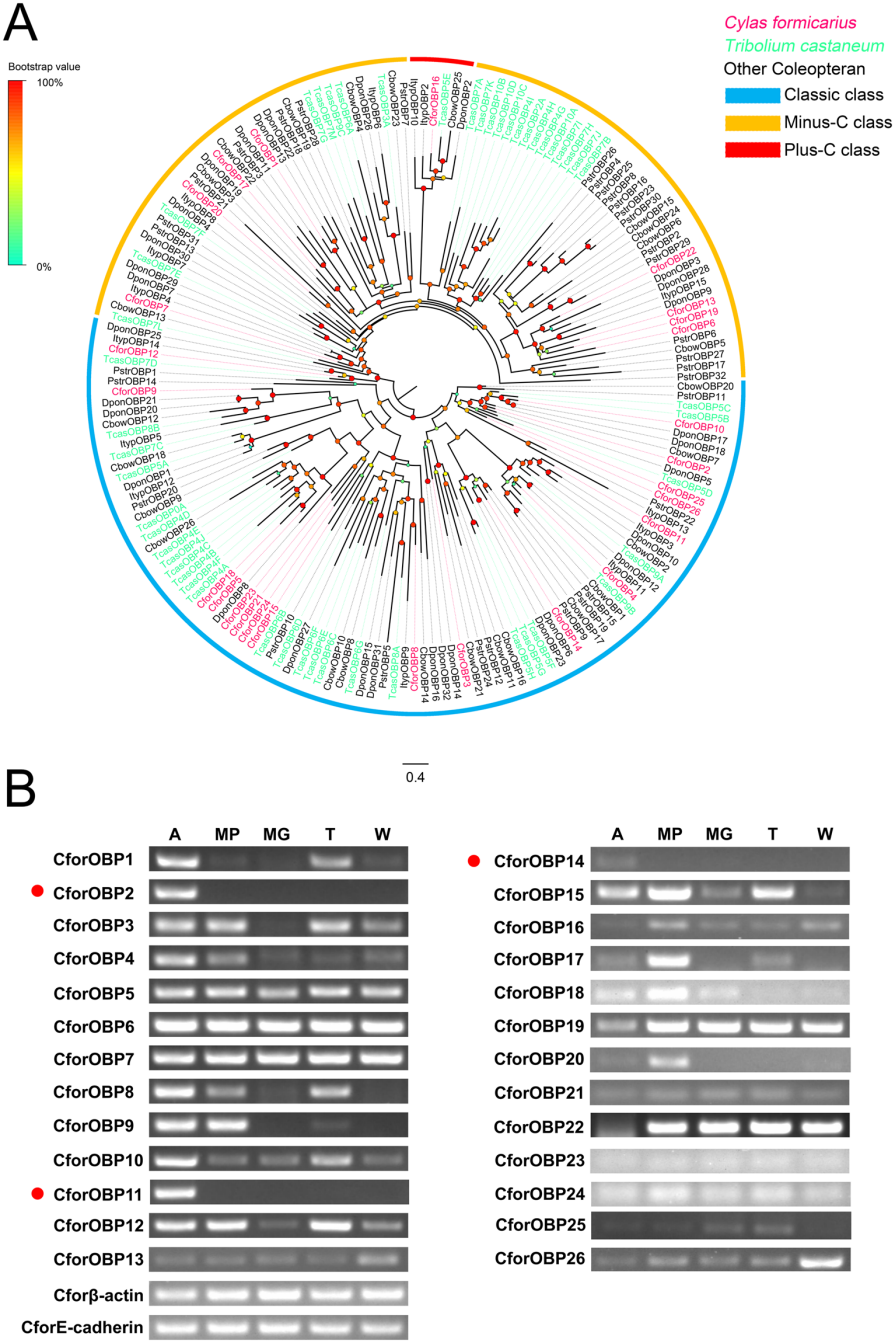
(**A**) Phylogenetic analysis of OBPs. Classic OBPs from *C. formicarius* and other coleopteran species form a clade labeled in red. Labeled in orange are Minus-C OBPs from *C. formicarius* and other coleopterans, and labeled in blue are Plus-C OBPs. Species abbreviations: Cfor, *Cylas formicarius*; Tcas, *Tribolium castaneum*; Ityp, *Ips typographus*; Dpon, *Dendroctonus ponderosae*; Cbow, *Colaphellus bowringi*; Pstr, *Phyllotreta striolata*. Branch support (circles at the branch nodes) was estimated using an approximate likelihood ratio test based on the scale indicated at the top left. Bars indicate branch lengths in proportion to amino acid substitutions per site. (**B**) Transcriptional profiles of putative *C. formicarius* OBPs in different body parts determined using semi-quantitative RT-PCR. The OBPs expressed specifically in antennae are labeled with red dots. Two reference genes, β-actin (Cforβ-actin) and E-cadherin (CforE-cadherin), were used as internal references to test the integrity of each cDNA template. Abbreviations: A: antenna; MP, mouthparts; MG, midgut; T, tarsus; W, wing.

Analysis of the *C. formicarius* antennal transcriptomes identified 26 candidate OBP transcripts, which matched with insect pheromone/OBP domains. All but four of these CforOBP transcripts (CforOBP17, 24, 25 and 26) had a complete ORF and possessed signal peptides. Insect OBPs can be classed into subfamilies based on the presence of cysteine residues, including Classic OBPs, Plus-C OBPs, and Minus-C OBPs^53^. Multiple amino acid sequence alignments showed that 18 CforOBPs with six highly conserved cysteine residues belonged to the Classic class, one belonged to the Plus-C class (CforOBP16), with 4–6 additional cysteines and one characteristic proline, and the remaining CforOBPs belonged to the Minus-C class, with a loss of two conserved cysteines (C2 and C5) (see Supplementary Figure S1). Phylogenetic analysis of CforOBPs with coleopteran sequences indicated that these CforOBPs segregated into the Classic, Plus-C, and Minus-C OBP sub-families (Fig. 7A).

The CforOBP transcripts displayed different patterns of tissue distribution and abundance. Only three transcripts (CforOBP2, 11 and 14) were almost exclusively transcribed in the antennae, while three transcripts (CforOBP8, 12 and 15) were abundant in the main insect olfactory and gustatory organs, i.e. antennae, mouthparts or tarsi. OBPs transcripts appear to be abundant in all or several body parts (Fig. 7B). RPKM data showed that four OBPs (CforOBP5, 10, 11 and 14) were expressed significantly higher in the male antennae and five OBPs (CforOBP15, 16, 17, 19 and 20) were significantly higher expressed in female antennae.

### Chemosensory proteins

**Figure 8:**
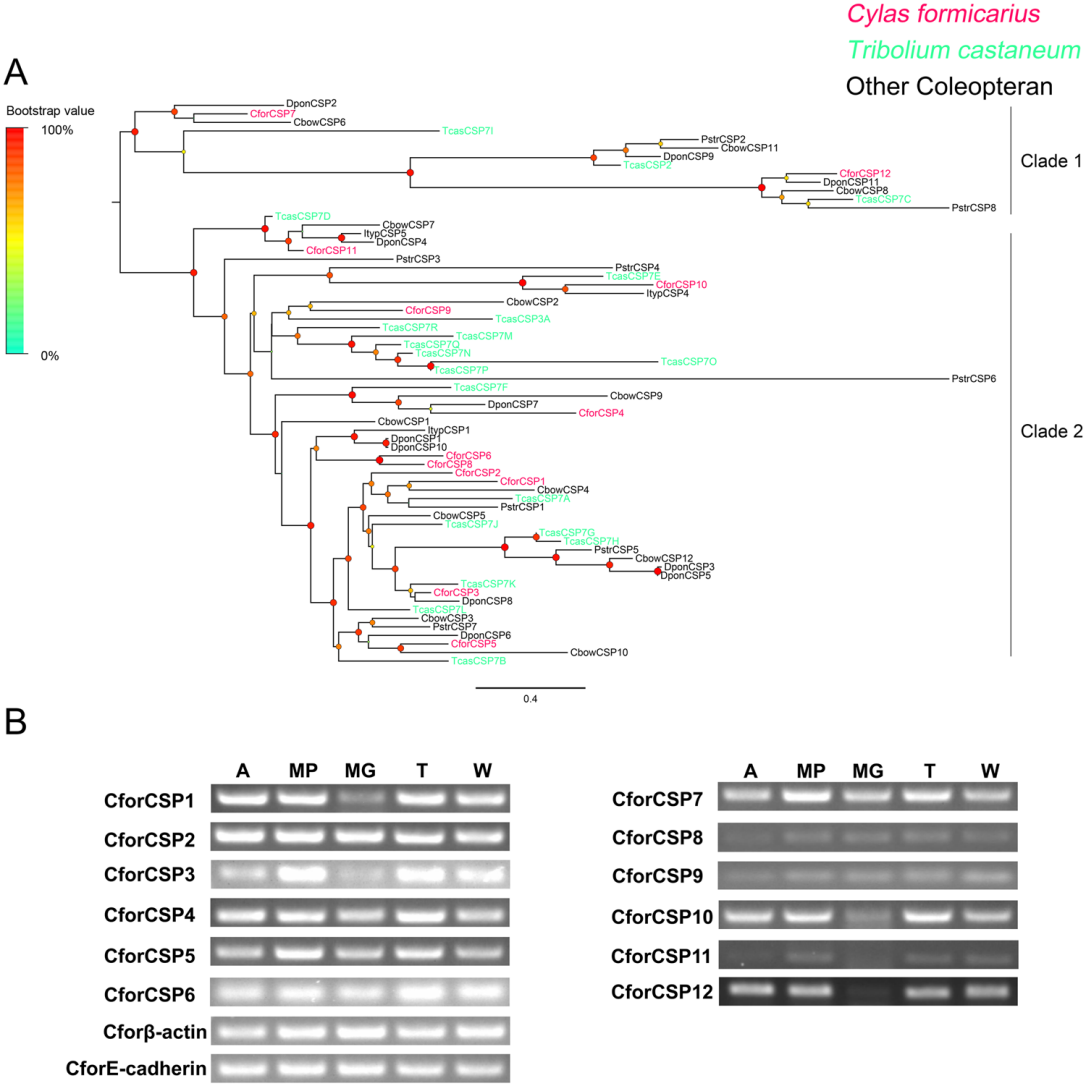
(**A**) Phylogenetic analysis of CSPs. Species abbreviations: Cfor, *Cylas formicarius*; Tcas, *Tribolium castaneum*; Ityp, *Ips typographus*; Dpon, *Dendroctonus ponderosae*; Cbow, *Colaphellus bowringi*; Pstr, *Phyllotreta striolata*. Branch support (circles at the branch nodes) was estimated using an approximate likelihood ratio test based on the scale indicated at the top left. Bars indicate branch lengths in proportion to amino acid substitutions per site. (**B**) Transcriptional profiles of putative *C. formicarius* CSPs in different body parts determined using semi-quantitative RT-PCR. Two housekeeping genes, β-actin (Cforβ-actin) and E-cadherin (CforE-cadherin), were used as internal references to test the integrity of each cDNA template. Abbreviations: A: antenna; MP, mouthparts; MG, midgut; T, tarsus; W, wing.

In total, 12 CSP encoding candidates were identified in the antennal transcriptomes, which were matched up with the OS-D domains. Among these CforCSPs, 8 contained a complete ORF and a signal peptide. Alignments of the amino acid sequences of *C. formicarius* CSP revealed the presence of a highly conserved four-cysteine profile (see Supplementary Figure S2). The phylogenetic tree of CforCSPs formed two lineage-specific clades, including clade 1 (2 CSPs) and clade 2 (10 CSPs) (Fig. 8A). Semi-quantitative RT-PCR analysis revealed that all CforCSPs were present in all or several body parts, and no candidate was enriched in the antenna (Fig. 8B).

## Discussion

Investigations on the molecular mechanisms of olfaction in *C. formicarius* provide insight into chemoreception and could ultimately lead to the identification of new targets for olfactory disruption and development of safer pest control strategies. We sequenced and analyzed the transcriptome of male and female *C. formicarius* antennae and identified and analyzed expression patterns of a set of novel chemosensory genes including 54 ORs, 11 GRs, 15 IRs, 3 SNMPs, 26 OBPs and 12 CSPs. Our results will be useful for functional characterization of olfaction genes in *C. formicarius*.

Based on phylogenetic tree analysis, the *C. formicarius* Orco (CforOR1) grouped with other coleopteran Orcos, but revealed a large species-specific expansion of *C. formicarius* ORs distributed in the previously defined coleopteran OR subfamily 7a that differ from other coleopterans^22^. This may reflect that *C. formicarius* inhabits a different ecological niche than the other coleopterans. Generally, sexually dimorphic expression of ORs in the antennae, indicate possible pheromone receptors contributing to facilitation of sexual behaviors. Typically, Lepidoptera sex pheromones are produced by females and they affect males. Several moth sex pheromone ORs have been functionally characterized, and most are expressed at higher levels in the male antennae^54–56^. *C. formicarius* females release the sex pheromone, (Z)-3-dodecen-1-ol (E)-2-butenoate, and elicit strong male attraction^39, 40^. Here, we found that four male-biased CforORs (CforOR4, 47, 52, and 54) reside in different clades, suggesting that the sex pheromone receptors might be screened from these candidates, according to the lock-and-key mechanism as they are activated by single pheromone components^57^. Additionally, ORs expressed biasedly in the female antennae are predicted to function in egg-laying-related odorant detection; ORs expressed evenly in the male and female antennae are predicted to function in general odorant perception^58, 59^. Therefore, we hypothesize that some or all of female-biased CforORs (CforOR13, 20, 23 and 24) appear to be involved in female specific behaviors i.e. finding plant hosts for oviposition, while remaining ORs with equal in the female and male antennae might be dedicated to general odorant detection or others.

The GR family of insect chemoreceptors includes receptors for CO_2_, D fructose, sucrose, bitter, and other receptors^60^. Gustatory receptors perceive essential nutrients whose chemical structures remain constant such as sugars and CO_2_ receptors, thus CO_2_ and sugar receptor genes are highly conserved among insects^61–63^. A total of 11 GR transcripts were identified in the *C. formicarius* antennal transcriptome dataset. The GR family in *C. formicarius* includes two putative CO_2_ receptors (CforGR21a and 63a), which were the orthologous genes of the GR21a/GR63a CO_2_ receptor, and two sugar receptors (CforGR43a.1 and 43a.2), share the same clade with DmelGR43a, a receptor of fructose as the nutrient sensor in the Drosophila brain^50^. We suggest that these GRs potentially have similar functions.

IR is another chemosensory receptor family that has been characterized in *D. melanogaster* ^52^. IRs are a conserved family and function as chemoreceptors for the detection of a variety of chemical molecules^9–11, 14, 64, 65^. Phylogenetic analysis indicated two co-receptors (IR25a and IR8a), five antennal IRs (IR40a, IR64a, IR68a, IR76b, and IR93a) in *C. formicarius* that have orthologs in both *D. melanogaster* and *T. castaneum*. The antennal IRs in *D. melanogaster* have been confirmed by functional studies. IR40a is required for response to the insect repellent DEET^10^, and IR64a is acid sensitive^14^. IR76b is co-expressed with IR41a to mediate long-range attraction to odor^9^, and IR93a acts with different combinations of other IRs to mediate physiological and behavioral responses to both temperature and moisture cues^66^. These IRs orthologs in *C. formicarius* might have similar sensory functions. But the function(s) of IR68a remain uncharacterized. We found CforIR68a and CforIR75s had male-biased expression in antennae and are likely to play a role in the perception of female pheromones.

In *D. melanogaster*, SNMP1 is expressed specifically in pheromone-sensitive ORNs, and is responsible for the sensitivity of these neurons to cVA stimulation^7, 8, 67^. In the present study, two SNMP subfamilies (both SNMP1 and SNMP2) were obtained from *C. formicarius* antennal transcriptomes. As expected, SNMP1 homologs (CforSNMP1) presented a clear antenna-predominant expression, while SNMP2 homologs (CforSNMP2a and 2b) were not restricted to the antennae. Additionally, RPKM results showed that CforSNMP1 is the only SNMP gene to have high expression in the antennae compared to the others suggesting that CforSNMP1 may play a role similar to its homolog in *D. melanogaster*.

OBP are commonly regarded as solubilizers and carriers of odorants and sex pheromones^68^. Additionally, OBPs may contribute to the sensitivity of the olfactory system^69–72^. They are not restricted to chemosensory tissues and may participate in other non-sensory functions^68^. We found that most CforOBPs were distributed in all examined body parts, and only three classic OBPs (OBP2, 11 and 14) were present exclusively in the antennae, suggesting that these participate in olfactory sensory functions. In the antennal-specific OBPs, CforOBP14 displayed male-biased expression that may play a role in sex pheromone perception. In some cases, CSPs as well as OBPs act as carriers of odorant molecules^73–76^. However, in our RT-PCR analysis, these CSPs were abundant in all body parts examined, suggesting that, in *C. formicarius*, these CSPs could be also be involved in non-sensory functions.

## Conclusion

We obtained substantial molecular information on the combined male and female antennal transcriptome of *C. formicarius* using high-throughput sequencing technology. The goal was to identify gene families involved in odorant detection. Based on the transcriptomic analysis, a repertoire of 54 ORs, 11 GRs, 15 IRs, 3 SNMPs, 26 OBPs and 12 CSPs were identified and further analyzed for their expression profiles. Our results directly provide a foundation for advanced functional studies of these olfactory genes in *C. formicarius*.

## Methods

### Insect rearing and collection

Sweetpotato weevils were originally collected in infested sweet potato fields in Xinhui County, Jiangmen City, Guangdong Province, China (E113°13′, N22°25′). The collected weevils were reared at the Institute for Management of Invasive Alien Species, Zhongkai University of Agriculture and Engineering. They were fed commercial sweet potato and kept in a climatic chamber at 28 °C ± 1 °C, 80% relative humidity, and a 16 h: 8 h light dark photoperiod. After 5–6 generations, 3–4 week-old adults, prior to reproduction, were used for experiments.

### RNA isolation and sequencing

Antennae of 200 males and 200 females were hand-dissected, flash frozen in liquid nitrogen, and then crushed with a hand mortar. Total RNA was isolated using TRIzol reagent (Invitrogen, USA) from male antennae and female antennae individually according to manufacturer instructions. RNA samples were treated with DNase (Qiagen, Germany) and then purified with RNeasy Mini Kit (Qiagen, Germany). The quality and concentration of total RNA were examined using Qubit^@^2.0 Fluorometer (Invitrogen, Life Technologies), and RNA integrity was further confirmed using an Agilent 2100 Bioanalyzer (Agilent Technologies, Palo Alto, CA, USA).

Paired-end cDNA libraries were generated from purified RNA (0.5 μg of each sample) using TruSeq RNA Sample Preparation Kit v2 (Illumina Inc., San Diego, CA) according to Illumina instructions and sequenced on the Illumina HiSeq. 4000 platform. The raw sequence transcriptome data from the female and male antennae libraries were deposited in the NCBI Short Read Archive (SRA) database as BioProject Accession Number SRP067907.

### *De novo* transcriptome assembly and gene annotation

Within the combined female and male antennae libraries, raw data (raw reads) of fastq format were first processed to remove unknown (poly-N) or low-quality sequences and adaptor sequences using FASTX-Toolkit (http://hannonlab.cshl.edu/fastx_toolkit/), and then assembled into unigenes using the Trinity pipeline (ver. r2013-02-25) with the default assembly parameters. All unigenes were annotated using BLASTx search against with a cutoff E-value of 10^−5^ the following databases: the non-redundant protein sequence (Nr), non-redundant nucleotide (Nt), Pfam, Clusters of Orthologous Groups (KOG/COG), Swiss-Prot, Kyoto Encyclopedia of Genes and Genomes (KEGG) and Gene Ontology (GO) databases, according to the highest sequence similarity.

### Chemosensory gene identification

Identification of putative *C. formicarius* chemosensory gene families using both BLASTx searches in Nr and tBLASTn searches using known sequences as queries. *C. formicarius* chemosensory genes were also used as queries to identify additional genes (tBLASTx and BLASTp). Repetitions were completed until no new candidates were identified. The open reading frames (ORFs) of putative chemosensory genes transcripts were identified using the ORF finder tool (http://www.ncbi.nlm.nih.gov/gorf/gorf.html), and further confirmed using the SMART BLAST searching result according to the best matches with well-studied reference species. Definitive protein domains (e.g. transmembrane domains, signal peptides, secondary structures, etc.) in chemosensory genes were predicted by queries against InterPro using the InterProScan tool plug-in in Geneious (ver. 9.1.3.)^77^.

### Alignment and phylogenetic analyses

Amino acid sequences were aligned using the MAFFT alignment tool plug-in in Geneious (ver. 9.1.3.) (E-INS-I parameter set)^78^. Phylogenetic relationship was deduced using maximum likelihood analysis with FastTree2 (JTT substitution model, 1000 bootstrap replications)^79, 80^ and subsequently viewed and graphically edited in FigTree v1.4.2 (http://tree.bio.ed.ac.uk/software/figtree). Incomplete transcripts lacking sufficient overlap in alignments and transcripts less than 180 amino acids in length (except for the OBPs where full-length transcripts are generally shorter than 200 amino acids) were excluded from phylogenetic analyses to ensure that the analysed transcripts corresponded to individual genes and to maintain greater accuracy in the analyses. The respective chemosensory genes families used for constructing phylogenetic trees are listed in Supplementary Table S2.

### Abundance estimation and differential expression analyses

Gene expression levels were assessed using the RSEM v1.2.8^81^ separately for the filtered reads from female and male antennae libraries, and their converted FPKM values (fragments per kilobase per million reads)^82^. Raw read counts data generated via RSEM were normalized using the Trimmed Mean of M-value normalization method^83^ and were used for differential expression analyses between female and male antenna using edgeR package (v3.4.2)^84^.

### Tissue expression analyses

Semi-quantitative RT-PCR was employed to investigate and compare the expression of SNMPs, OBPs and CSPs in different tissues including antennae, mouthparts, midgut, foreleg tarsus, and wings, to define the antenna-predominant candidates. Total RNA from the analyzed tissues was isolated using an RNeasy Mini kit (Qiagen, Germany), and cDNA was synthesized using PrimeScript RT reagent Kit (Takara, China). An equal amount of cDNA (100 ng) was used as the Semi-quantitative RT-PCR templates. Primers were designed using Primer3web (ver. 4.0.0) (http://primer3.ut.ee/) and are listed in Supplementary Table S3. PCR was performed under the following conditions: 95 °C for 2 min, followed by 35 cycles of 95 °C for 30 sec, 56 °C for 30 sec, 72 °C for 1 min, and a final extension for 10 min at 72 °C. To reach reproducibility, each semi-quantitative RT-PCR was repeated three times with two independently isolated RNA samples. The semi-quantitative PCR products were analyzed on 1.5% agarose gel electrophoresis. According to a previous study^85^, two housekeeping genes, β-actin (Cforβ-actin) and E-cadherin (CforE-cadherin) from *C. formicarius* antennal transcriptomes were used as the controls.

The expression profiles of ORs and antennal IRs were analyzed using quantitative real-time PCR (qPCR). First, RNA isolation and cDNA synthesis were performed on samples including 20 male or female antennae each and 10 whole insect bodies without antennae (male and female, ratio 1:1). qPCR was performed on a LightCycler 480 system (Roche Applied Science) in a reaction volume of 10 μl SYBR Green I Master mix (Roche Applied Science), 1 μl of each primer (0.5 μM), 2 μl (approximately 2.0 ng) of sample cDNA and 6 μl sterilized ultrapure H_2_O. The cycling parameters were as follows: denaturation at 95 °C for 5 min, followed by 45 cycles of 95 °C for 10 sec, and 60 °C for 20 sec. A melting curve analysis was then performed at 95 °C for 20 sec, 60 °C for 30 sec, and 95 °C for 30 sec in order to determine the specificity of primers. Negative controls without template were included in each reaction. For each gene, three biological replications (3 separate RNA extractions from samples) were performed with each biological replication measured in three technical replications. The results were analyzed using LightCycler 480 Gene Scanning Software. The comparative 2^−ΔΔCT^ method was used to calculate the relative expression levels of each gene^86^, with two housekeeping genes β-actin and E-cadherin as the reference genes. The comparative analyses of each target gene among different tissues were determined using a one-way nested analysis of variance (ANOVA) followed by Tukey’s honest significance difference (HSD) test using Prism 6.0 (GraphPad Software, CA). Values are presented as mean ± SE.

## Electronic supplementary material

Supplementary Information

Supplementary Dataset
