# Supplementary material for: Antennal transcriptome and expression analyses of olfactory genes in the sweetpotato weevil Cylas formicarius
Source: Sci Rep. 2017 Sep 11;7:11073. doi: 10.1038/s41598-017-11456-x (PMC5593998; doi:10.1038/s41598-017-11456-x)
Supplement: Supplementary file 1 — Supplementary Information [file 41598_2017_11456_MOESM1_ESM.doc]

**Supplementary Information for**

**Antennal transcriptome and expression analyses of olfactory genes in the sweetpotato weevil *Cylas formicarius***

Shu-Ying Bin, Meng-Qiu Qu, Xin-Hua Pu, Zhong-Zhen Wu*, Jin-Tian Lin*

Institute for Management of Invasive Alien Species, 314 Yingdong teaching building, Zhongkai University of Agriculture and Engineering, Guangzhou 510225, PR China

Email addresses:

**Shu-Ying Bin**: [binsuying@163.com](mailto:binsuying@163.com)

**Meng-Qiu Qu**: qumengqiu@163.com

**Xin-Hua Pu**: pu123xh@163.com

**Zhong-Zhen Wu**: zhongzhen_wu@163.com

**Jin-Tian Lin**: [linjtian@163.com](mailto:linjtian@163.com)


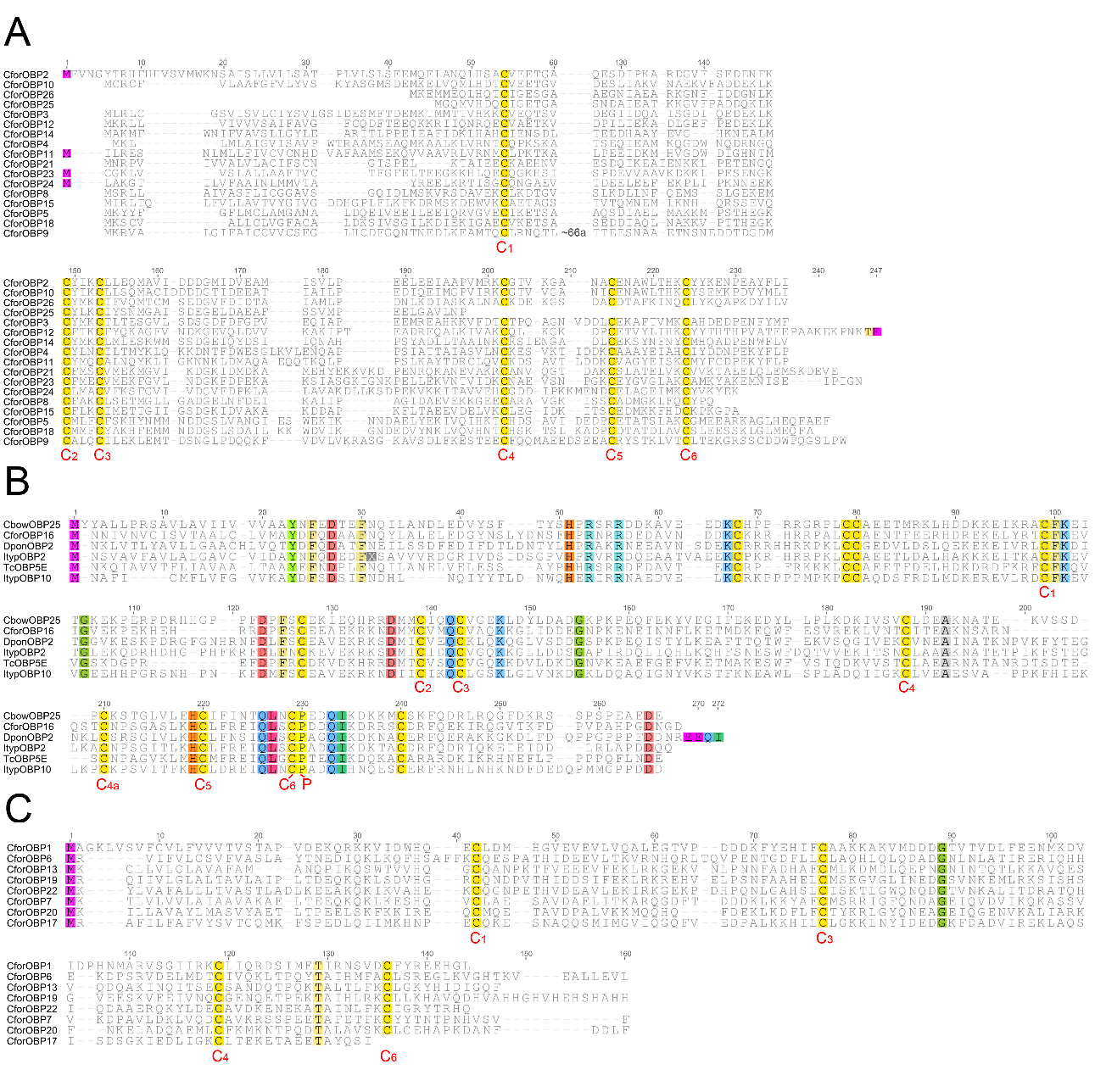


**Supplementary Figure S1**. Excerpts from the amino acid alignment showing the predicted Classic OBPs (A), Plus-C OBPs (B), and Minus-C OBPs (B).

**
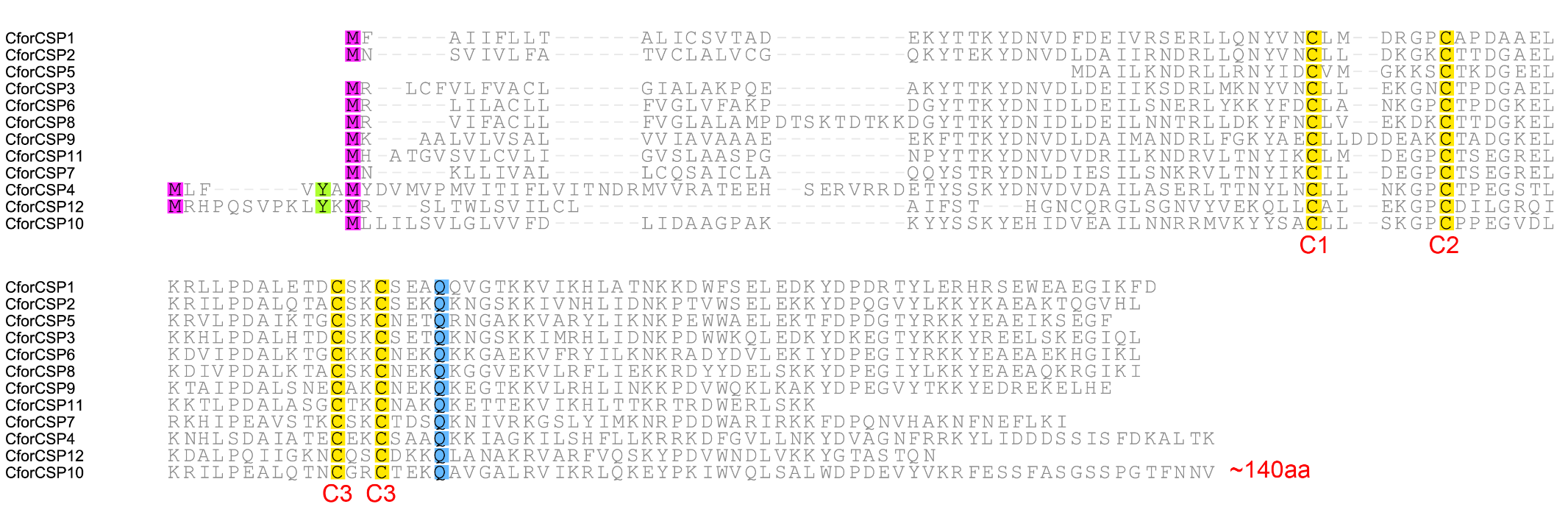
**

**Supplementary Figure S2.** Excerpts from the amino acid alignment showing the predicted CSPs.

**Supplementary Table S1. Overview of the sequencing and assembly process.**

|  | MA (male) | FA (female) |
| --- | --- | --- |
| Mean length (bp) | 150 | 150 |
| Total Raw Reads | 51,183,400 | 48,433,914 |
| Total Raw Bases | 7,677,510,000 | 7,265,087,100 |
| Total Clean Reads | 51,025,928 | 48,238,192 |
| Total Clean Reads Ratio (%) | 99.69 | 99.6 |
| Total Clean Bases | 7,653,889,200 | 7,235,728,800 |
| Total Clean Bases Ratio (%) | 99.69 | 99.6 |
| Total Adapter Reads | 155,552 | 193,852 |
| Total Adapter Reads Ratio (%) | 0.30 | 0.4 |
| Total Low Quality Reads | 1,920 | 1,870 |
| Total Low Quality Reads Ratio (%) | 0.00 | 0 |
| Clean Reads GC (%) | 41.89 | 41.58 |
| Clean Reads Q20 (%) | 96.73 | 96.72 |
| Clean Reads Q30 (%) | 91.81 | 91.83 |
| Combined Trinity assembly of the male and female antennal transcriptomes | | |
| Total Number | 66531 | |
| Min Length (bp) | 200 | |
| Max length (bp) | 25394 | |
| Mean Length (bp) | 1384 | |
| Unigene N50 | 2924 | |
| Unigene N90 | 506 | |
| Unigene GC (%) | 0.3987 | |

**Supplementary Table S2**. Amino acid sequences of *C. formicarius* and other insect species used in phylogenetic analyses.

| **Gene** | **Species** | **Annotation** | **Total genes** | **Selected genes** | **References** |
| --- | --- | --- | --- | --- | --- |
| ORs | *Cylas formicarius* | Transcriptomes | 54 | 54 | This study |
|  | *Tribolium castaneum* | Genomes | 111 | 92 | [1] |
|  | *Megacyllene caryae* | Transcriptomes | 57 | 48 | [2] |
|  | *Ips typographus* | Transcriptomes | 43 | 26 | [3] |
|  | *Dendroctonus ponderosae* | Transcriptomes | 49 | 32 |
|  | *Colaphellus bowringi* | Transcriptomes | 43 | 31 | [4] |
| GRs | *Cylas formicarius* | Transcriptomes | 11 | 11 | In this study |
|  | *Drosophila melanogaster* | Genomes | 68 | 68 | [5] |
|  | *Tribolium castaneum* | Genomes | 181 | 181 | [6] |
| iGluRs/IRs | *Cylas formicarius* | Transcriptomes | 15 | 15 | This study |
|  | *Drosophila melanogaster* | Genomes | 80 | 80 | [7] |
|  | *Tribolium castaneum* | Genomes | 35 | 35 |
|  | *Ips typographus* | Transcriptomes | 7 | 7 | [3] |
|  | *Dendroctonus valens ponderosae* | Transcriptomes | 15 | 15 |
| SNMPs | *Cylas formicarius* | Transcriptomes | 3 | 3 | This study |
|  | *Drosophila melanogaster* | Genomes | 2 | 2 | [8] |
|  | *Tribolium castaneum* | Transcriptomes | 6 | 6 |
|  | *Ips typographus* | Transcriptomes | 3 | 2 | [3] |
|  | *Dendroctonus ponderosae* | Transcriptomes | 3 | 2 |
|  | *Dendroctonus valens* | Transcriptomes | 4 | 3 | [9] |
| OBPs | *Cylas formicarius* | Transcriptomes | 26 | 26 | This study |
|  | *Tribolium castaneum* | Transcriptomes | 50 | 50 | [10] |
|  | *Ips typographus* | Transcriptomes | 15 | 14 | [3] |
|  | *Dendroctonus ponderosae* | Transcriptomes | 31 | 31 |
|  | *Colaphellus bowringi* | Transcriptomes | 26 | 26 | [4] |
|  | *Phyllotreta striolata* | Transcriptomes | 32 | 32 | [11] |
| CSPs | *Cylas formicarius* | Transcriptomes | 12 | 12 | This study |
|  | *Tribolium castaneum* | Transcriptomes | 20 | 20 | [10] |
|  | *Ips typographus* | Transcriptomes | 6 | 3 | [3] |
|  | *Dendroctonus ponderosae* | Transcriptomes | 11 | 11 |
|  | *Colaphellus bowringi* | Transcriptomes | 12 | 12 | [4] |
|  | *Phyllotreta striolata* | Transcriptomes | 8 | 8 | [11] |

**References**

1. Engsontia, P. *et al.* The red flour beetle's large nose: an expanded odorant receptor gene family in *Tribolium castaneum*. *Insect Biochem. Mol. Biol.* 38, 387-397 (2008).
2. Mitchell, R.F. *et al.* Sequencing and characterizing odorant receptors of the cerambycid beetle *Megacyllene caryae*. *Insect Biochem. Mol. Biol.* 42, 499-505 (2012).
3. Andersson, M.N. *et al.* Antennal transcriptome analysis of the chemosensory gene families in the tree killing bark beetles, *Ips typographus* and *Dendroctonus ponderosae* (Coleoptera: Curculionidae: Scolytinae). *BMC Genomics* 14, 198 (2013).
4. Li X.M. *et al.* Candidate chemosensory genes identified in *Colaphellus bowringi* by antennal transcriptome analysis. *BMC Genomics* 16, 1028 (2015).
5. Robertson H.M., Warr C.G. & Carlson J.R. Molecular evolution of the insect chemoreceptor gene superfamily in *Drosophila melanogaster*. *Proc. Natl. Acad. Sci.* 1002, 14537-14542 (2003).
6. Richards S, *et al*. The genome of the model beetle and pest *Tribolium castaneum*. *Nature* 452, 949-955 (2008).
7. Croset, V. *et al.* Ancient protostome origin of chemosensory ionotropic glutamate receptors and the evolution of insect taste and olfaction. *PLoS Genet.* 6, e1001064 (2010).
8. Vogt, R.G. *et al.* The insect SNMP gene family. *Insect Biochem. Mol. Biol.* 39, 448-456 (2009).
9. Gu, X.C., Zhang, Y.N., Kang, K., Dong, S.L. & Zhang, L.W. Antennal Transcriptome Analysis of Odorant Reception Genes in the Red Turpentine Beetle (RTB), *Dendroctonus valens*. *PloS one* 10, e125159 (2015).
10. Dippel, S. *et al.* Tissue-specific transcriptomics, chromosomal localization, and phylogeny of chemosensory and odorant binding proteins from the red flour beetle *Tribolium castaneum* reveal subgroup specificities for olfaction or more general functions. *BMC Genomics* 15, 1141 (2014).
11. Wu, Z., Bin, S., He, H., Wang, Z., Li, M. & Lin, J. Differential Expression Analysis of Chemoreception Genes in the Striped Flea Beetle *Phyllotreta striolata* Using a Transcriptomic Approach. *PloS one* 11, e153067 (2016).

**Supplementary Table S3.** Primers used in qPCR.

| **Gene Name** | **Forward Primer (5'-3')** | **Reverse Primer (5'-3')** | **TM (℃)** | **Product size (bp)** |
| --- | --- | --- | --- | --- |
| Reference genes |  |  |  |  |
| Cforβ-actin | TCTTGGGTATGGAAGCGTGT | GCGGTGATTTCCTTCTGCAT | 59 | 160 |
| CforE-cadherin | GTCGCATCGTGATTGAGCTT | TGGTAAAGTCTCCGCCTTGA | 59 | 153 |
| ORs |  |  |  |  |
| CforOR1 | ACGTCCTATTCTCCCTGCTG | GCTAGATGGCGCTTTGAACA | 59 | 179 |
| CforOR2 | CTCTACGAATATGTGGCGGC | GTTGTTGATGTTCCTGGGCA | 59 | 175 |
| CforOR3 | GTTCTTCTTGGGATGGAGCG | GAGTTGAGTGTCATTTCGCCA | 59 | 186 |
| CforOR4 | CACAGACAAGCACCTCACAC | TAAGACATCCTCCGGTGACC | 59 | 193 |
| CforOR5 | AAGACGCCGACCAAATGTTC | GCTCGTCCTCCATATACCCC | 59 | 153 |
| CforOR6 | CTGGCGTCCGAAGAACAATT | ACAATCGCAAAGCACACGTA | 59 | 169 |
| CforOR7 | TGTTCGTCCTTGTCTCCTCC | ACTAGTCACACCGAGGCAAA | 59 | 227 |
| CforOR8 | CATCAGCGGGAAGAAGAAGC | GATCACCAGCGCGAATGTAG | 59 | 153 |
| CforOR9 | TCGTATTTGCGGTGGTTGAA | TCGAACGCCAGTAAGAGTGT | 59 | 154 |
| CforOR10 | CCGGAAAGACGCCAACTATG | TGAATCATACCCAGGCTCCC | 59 | 210 |
| CforOR11 | GCATCCAATTCGCCATGTCT | GTATTTCCCTCTCCTCGGCA | 59 | 168 |
| CforOR12 | GTTGAAGGGCTTGGATGGTC | GTTCGACGCCTCCAATGATC | 59 | 176 |
| CforOR13 | CAGCCAAGCCGTTCATCTAC | GTTCGGAAATCAGACGCCTC | 59 | 240 |
| CforOR14 | TGTTAGTACAGCTGGCGGAT | TGCTATTGTCCAGAGGCAGT | 59 | 160 |
| CforOR15 | TCGCCAATACCGACCAAGTA | TTGTGTCCTCCGAGAACCTC | 59 | 178 |
| CforOR16 | GGTCTTTGGTTTGAGCGGAG | GTGTTCGCCATGAGAGTGAC | 59 | 170 |
| CforOR17 | TTATAGGGCAGCTCGTCGAA | AAACACCTCCGCCATGACTA | 59 | 184 |
| CforOR18 | AGAGTTCGGGATTAGCGGAC | GAAACTCCGGCCTCCAAATC | 59 | 157 |
| CforOR19 | GCAATCTTCGCTGCAAATCG | CCAACCGCTGTCCCATATTG | 59 | 190 |
| CforOR20 | TATTTGGCGCTCACCTACCA | ATTCGTTCTCATCGTCGGGT | 59 | 169 |
| CforOR21 | CTGCTTATGTATCGGCGTGG | TAATTGTTGGTGTTGGGCGC | 59 | 166 |
| CforOR22 | GGGCTGGACATCAAACGAAA | AAGCCGCTTTCATCATCGTC | 59 | 203 |
| CforOR23 | ATCCGTCGTCACCTTCACTT | TATAGACAATGCCACGCCGA | 59 | 199 |
| CforOR24 | TTCTGACCGCCCTGTATAGC | ACTGCGAGTGATTTCAAGCC | 59 | 175 |
| CforOR25 | GATGACGTCGCCCAACTTTT | TCGACAGATACAGAGGACGC | 59 | 215 |
| CforOR26 | ATGGCCGCTGATATTCGAGA | GGGTCTATTCAAGGCTCGGA | 59 | 223 |
| CforOR27 | CCTGCTCACTGAAGCCATTG | GCAGAGGTTTTACTAGCGCC | 59 | 227 |
| CforOR28 | TCATGATCGCCGGTGGTATT | TGTTGGTTTGGCAGAGAAGC | 59 | 240 |
| CforOR29 | TCGGTCCCAAAGAGAACGTT | TGAAATTCCGACTGCAAGGC | 59 | 236 |
| CforOR30 | TTCGCAAAATCCACAGGCAA | CTGTCGAAGTTTCTGAGCCG | 59 | 232 |
| CforOR31 | GAGGCCTTTAACGGTGCATT | AAAAGTTTGTCCTGCCCGAC | 59 | 174 |
| CforOR32 | GGAGCCATTAACGCCGAAAT | TGTTTATGCCCCTGTGCTCT | 59 | 166 |
| CforOR33 | GACCCTATGCCAGTCGAAGA | TTGGCGGTATTGTGCGAAAA | 59 | 228 |
| CforOR34 | GCGCTGCTCTACTACGTCTA | AAAATGCCGAAAACGCTTGC | 59 | 208 |
| CforOR35 | GTTCCCAGCTTTAACCCGTG | CTGCCGTCTCACAATTCCTG | 59 | 213 |
| CforOR36 | GCCCATAAGCTTCTCGTTCG | AGTATGTCGAGTTGTGCCGA | 59 | 156 |
| CforOR37 | CATGGTGGCCTGCGAAATAT | TCTAAGGTGCTCTAGTCGCG | 59 | 161 |
| CforOR38 | CGCCGTCTCGATTCAATTCA | TTTTGTGCGCGAAGGATCAT | 59 | 213 |
| CforOR39 | TCGCCGCTTTATTGTTCCAG | TAAGGGCATCTGACAGCGAA | 59 | 227 |
| CforOR40 | GGCGAATACCCGTTCAATCC | CATTTTCTCAGGGCCACGTC | 59 | 224 |
| CforOR41 | TACTACAGCAATGCCCACGA | ATGGTGGTCTTCATCCCCTG | 59 | 250 |
| CforOR42 | CAGCCAAGCCGTTCATCTAC | GTTCGGAAATCAGACGCCTC | 59 | 240 |
| CforOR43 | GACCGAGAAAGTGTTTGGCA | CTGTTGCTGGAGTCTACCCA | 59 | 249 |
| CforOR44 | GCCGATCAGAGTTCGCAATT | GCTTGACAATCGAAACCCCA | 59 | 172 |
| CforOR45 | TATCGCTGACCCTCGATGAG | TATCAAATTTCTGCCGCCGG | 59 | 198 |
| CforOR46 | GCTTGCGGGTCACAATGTAA | AGTGTCATAGGCCCAAACGA | 59 | 240 |
| CforOR47 | TTATCGGACGCTTTGCATGG | TCCACGCTCCTATCGCTATG | 59 | 182 |
| CforOR48 | GGCTGCTCTTTCTTTCGCAT | TCGGGTTTCTGCACTTGAGA | 59 | 220 |
| CforOR49 | TAAACAGTCCAACTTCGGCG | CAGGGCTAACAACATCGACG | 59 | 196 |
| CforOR50 | TGGATTCTCATCGAGCAGCT | TGTGAGGCTTTCGTTTGCAT | 59 | 177 |
| CforOR51 | GTGTGTTATTACGGGCAGCA | AACTACTCCCATACCGGCAG | 59 | 168 |
| CforOR52 | AGATCCGCTAGCAGTAGACG | CGGTTTGTAGGCCATGTAGC | 59 | 154 |
| CforOR53 | CCTTGGAATTCGGTTTGGGG | CGCTTCCGGTTCTATCTCCT | 59 | 203 |
| CforOR54 | CAGGGCGTTAACCAATGTGT | GCTCGTACCATTTGCTCTCG | 59 | 232 |
| Antennal IRs |  |  |  |  |
| CforIR25a | CTCAGGGACCTCACCAAACT | GTCCTGATACACCGCGTCTA | 59 | 216 |
| CforIR8a | AGTCTGGCTGAGTATCGTGG | ATATGCGGCCACCAATGTTC | 59 | 226 |
| CforIR76b | GCTTTCTCGCCAACCTTCAA | AACTGTGGACGGCAAATTCC | 59 | 244 |
| CforIR40a | CGGTTGCCCTTTTATCGAGG | GTTGTCCTCCGTCGCTTTAG | 59 | 196 |
| CforIR41a | TAAGCAACGTGGAGCAATGG | ATCTGTTGGAGGGTTTCGGT | 59 | 204 |
| CforIR64a | ATGACGTTTACAATCCCGCG | TGGATGTCTCTGTCGCTCAA | 59 | 217 |
| CforIR68a | ACGTCATTTTGGTGGCAGAC | GCCATTCCCAGGTTTCTTGG | 59 | 200 |
| CforIR75c | GAAAGCAAGCTCAGAGTCGG | AAGGTCTTCGCTATCAGGGG | 59 | 227 |
| CforIR75q.1 | AACCTTTACTCCTGCCGACA | GCACCTAAGGCACAAACGAA | 59 | 177 |
| CforIR75s | TATGTCAACAAGGGGCGTCT | CTCCAACCTGTAGCCTCGAT | 59 | 186 |
| CforIR93a | CCTGCCTCCTTACTTTGGGA | TCTTCCAGCACTTGTCCCTT | 59 | 177 |
| SNMPs |  |  |  |  |
| CforSNMP1 | ATCTACGAGAACCCACCGTC | TATCGTCTTGCTGGGAGTCC | 59 | 210 |
| CforSNMP2a | CGAAACTTGCAACAGGGTGA | CGCGTTGATTCCCAGATACG | 59 | 154 |
| CforSNMP2b | TCGTTCTACTGGAGCCGATC | CTTGATTGCGTCCAGGTAGC | 59 | 182 |
| OBPs |  |  |  |  |
| CforOBP1 | CGACGACAAGTTCTACGAGC | TCGACCGAGTTTCTGATGGT | 59 | 204 |
| CforOBP2 | TGGCCAATCAACTTCACAGC | CTTCCAGTTCTTCAGGCAGC | 59 | 204 |
| CforOBP3 | AAACCATGATGACCACCGTG | TGTCGCTTAACACTCCCGAT | 59 | 156 |
| CforOBP4 | TGAAACTGTTGATGCTGGCG | CGTTTCGGTCTTGGTTCCAG | 59 | 183 |
| CforOBP5 | GAATGCGCTGGACCAAGAAA | TTTCCCATGATTCGATGCCA | 59 | 230 |
| CforOBP6 | AGACGCGGATGGAAACCTTA | GCGACAAACAGGCAAACATG | 59 | 155 |
| CforOBP7 | TCGACGCAGAGCTCATAACA | GACTGGACCTTTTGACAGCG | 59 | 216 |
| CforOBP8 | AGCTATCGTTCCGGACTTGT | AGGTGCCTTGGGTGTTGTAA | 59 | 197 |
| CforOBP9 | TGTTACCAAAGGCACCAACG | TGGAATTTGTCTCAGCAGCG | 59 | 164 |
| CforOBP10 | GGGTCGACGAAAGCTTGATC | TCCGACTACTGTTCCGCATT | 59 | 215 |
| CforOBP11 | GCTTTGCCAGAGGAAATCGA | TGTCATCGAGAGTCACAGCT | 59 | 229 |
| CforOBP12 | CGTCGGCTTCTGTCAGGAT | TTTTCGCTTTGACCACGTCC | 59 | 227 |
| CforOBP13 | GGTTTTATGTCTGGCGGTGG | TGGTCAGCGAAATTGTTGGG | 59 | 171 |
| CforOBP14 | GGAGGATCATGCTGCTTACG | AGATCTGCATAGGACGGGTG | 59 | 153 |
| CforOBP15 | TTCGTGTTGTTGGCGGTAAC | CCGTCGTTTCCATGATGCAT | 59 | 211 |
| CforOBP16 | GTTCGAATCAGTCCGGGAGA | ATCACCGTTGTCACCTGGAT | 59 | 247 |
| CforOBP17 | CATCCTCTGTCTGGGCAAGA | GTTTCTTCGGCGGTTTCCTT | 59 | 159 |
| CforOBP18 | GGATCTCTCAGTGACGCCAT | TAGGGCTCCAACTACGCAAA | 59 | 215 |
| CforOBP19 | AGTTGTGCATGTCGAAAGGG | AGAATGCTCGTGAACATGGC | 59 | 239 |
| CforOBP20 | AAAGATCCGCGAACAATGCA | CAACATCTCTGCTTGGTCGG | 59 | 220 |
| CforOBP21 | TGCAAAGCTGAACACAACGT | GTACCTTGCACATTGGCACA | 59 | 242 |
| CforOBP22 | GACCACCCTCAGAACCTCG | GGTGGCCTTTTCGTTTTCCT | 59 | 186 |
| CforOBP23 | ACAAGAATGCCAGGGAAAGC | TGAGACTTCAGCATTGCACT | 59 | 250 |
| CforOBP24 | GTGTCTGATGGCATGTGTCT | ATCTCACCAGCCAGTTCACA | 59 | 198 |
| CforOBP25 | CGGGAGTAGAGATACCAGACG | ACAGCCCCTAATTCCTCTGG | 59 | 244 |
| CforOBP26 | CTGCAAGGATGAAAAGGGCA | TAACATCTGGGAAACAATGGCA | 59 | 150 |
| CSP |  |  |  |  |
| CforCSP1 | CGCTGAGTTGAAACGCCTAT | TCCCTTCAGCTTCCCATTCG | 59 | 200 |
| CforCSP2 | TCCGCAACGACAGACTTTTG | TCTCCAACTCGCTCCATACG | 59 | 207 |
| CforCSP3 | GCGATGAGGTTGTGTTTCGT | CTGGGGTGCAGTTTCCTTTC | 59 | 178 |
| CforCSP4 | TGTGATGGTGCCTATGGTCA | TTGCAAGAATGGCGTCAACA | 59 | 152 |
| CforCSP5 | GGGAAAGAAAAGCTGCACCA | TTTTCCAGTTCCGCCCACC | 59 | 165 |
| CforCSP6 | TCTCCTCTTCGTCGGATTGG | TCTCGGCACCTTTCTTCTGT | 59 | 233 |
| CforCSP7 | CGCAGAAGAACATCGTGAGG | GGGGAAATGATGTGAACGCT | 59 | 214 |
| CforCSP8 | CAGACACCTCCAAGACCGAT | TTGGAGCAGGCAGTTTTCAG | 59 | 196 |
| CforCSP9 | AAGGAGCTCAAGACTGCCAT | TCCTTCTCCCTGTCCTCGTA | 59 | 191 |
| CforCSP10 | GTACTCCTAGCGCGACTACC | ACCTAAAGCCCCTATGCCTC | 59 | 176 |
| CforCSP11 | ATGTTTGATGGACGAAGGCC | CTTTCCCAGTCTCTTGTGCG | 59 | 165 |
| CforCSP12 | GTCGGTGCCCAAACTTTACA | GTCTTTGATTTGCCTGCCCA | 59 | 184 |
